# Water Structure within Ternary Solvents Suggests Nanophase Formation

**DOI:** 10.1021/jacs.6c10066

**Published:** 2026-08-17

**Authors:** Binish Ashfaq, Chun-Ting Lin, Paul S. Cremer, Lauren D. Zarzar

**Affiliations:** † Department of Chemistry, 8082 The Pennsylvania State University, University Park, Pennsylvania 16802, United States; ‡ Department of Biochemistry and Molecular Biology, The Pennsylvania State University, University Park, Pennsylvania 16802, United States; § Department of Materials Science and Engineering, The Pennsylvania State University, University Park, Pennsylvania 16802, United States; ∥ Materials Research Institute, The Pennsylvania State University, University Park, Pennsylvania 16802, United States

## Abstract

Water’s unique solvating properties and its interactions with hydrophobic surfaces play crucial roles in various chemical processes ranging from self-assembly to phase separation. This study investigates the structure and hydrogen bonding of water in ternary solvent systems composed of water, a cosolvent (acetone, dimethylformamide, or dimethyl sulfoxide), and an oil (2-hexanone or 2-pentanone) to reveal how solvent organization changes upon oil addition. Using Raman spectroscopy coupled with multivariate curve resolution, we probe changes in hydrogen bonding as a function of ternary composition. Counterintuitively, replacing the cosolvent with oil while maintaining constant water levels reduces the volume of perturbed water within hydrophobic hydration shells. We also find a higher population of dangling hydroxyl groups upon oil addition. We propose that these results are indicative of the formation of nanoscale, oil-rich aggregates (nanophases) dispersed in water. This work offers insights into the role of hydrophobic hydration in ternary solvents, contributing to our understanding of nanophase formation, solvent behavior, and interfacial phenomena in complex mixtures.

## Graphical abstract

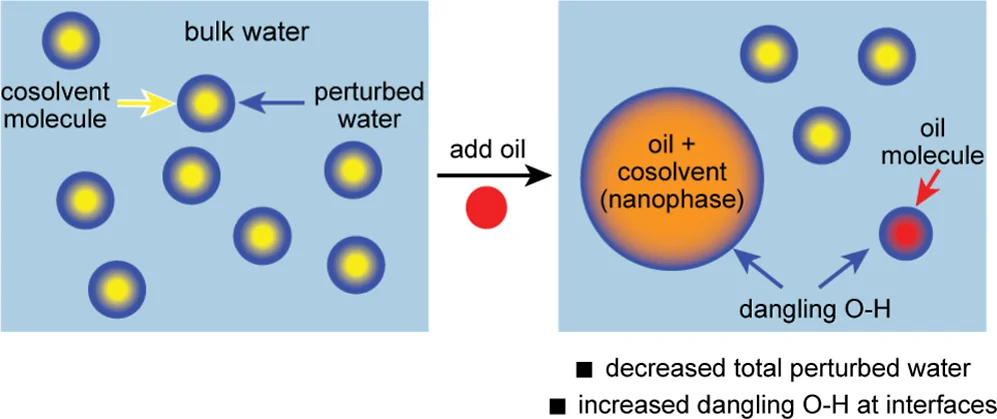

## Introduction

Water has unique solvating properties and exerts a notable influence on chemical processes, reactions, and molecular organization in solutions. In particular, water’s interactions with hydrophobic surfaces influence phenomena such as self-assembly,^1^ phase separation,^2^ hydrophobic effects,^3,4^ “on-water” and “in-water” chemical reactivity,^5,6^ protein folding,^7,8^ and the formation of ordered structures in colloids and membranes.^9,10^ These phenomena arise from water’s ability to form extensive hydrogen bonding networks that reorganize in response to variation in solutes, surface chemistry, and other external perturbations, producing effects that extend from the molecular scale to macroscopic material properties.

Early work by Frank and Evans introduced the “iceberg” model of hydrophobic hydration, proposing that water molecules form highly ordered, ice-like structures around apolar solutes.^11^ Later investigations revealed that although the hydrogen bonding network remains largely intact, the orientational and vibrational dynamics of water are significantly slowed near hydrophobic interfaces.^12–14^ Probing changes in the vibrational states of water as a function of solute composition provides insight into the interactions and arrangements of the molecules in solutions. Thus far, most experimental and theoretical studies that directly probe changes in water structure around hydrophobic moieties have focused on chemically minimal systems containing a single hydrophobic solute at low concentrations (≤0.5 M).^15,16^ However, biological and chemical environments typically involve multiple solutes of varying polarity and often higher concentrations or crowded environments. Even for commonplace ternary solvent mixtures, such as those containing water, oil, and cosolvent, the influence of water structure on solvent organization and solution properties is not well understood. Such ternary solvents have attracted attention due to their solubilization power, potential to serve as green substitutes for surfactant-based emulsions, and as replacements for organic solvents.^17^ Here, we investigate the structure and hydrogen bonding of water within ternary mixtures to shed light on how solution complexity may lead to molecular organization, nanoscopic phase formation, and influence solvent properties.

To probe changes in water structure, Raman spectroscopy in tandem with multivariate curve resolution (Raman MCR) has emerged as a robust vibrational spectroscopy method, enabling characterization of the molecular organization of water in the presence of hydrophobic solutes.^18^ Using Raman MCR, a measured Raman spectrum of a solution (water with solute) can be decomposed into a pure bulk water spectrum and a solute-correlated (SC) spectrum to reveal the organization and amount of “perturbed” water in the hydration shells of the solvent. Raman MCR has been successfully employed to investigate the structure of water in binary mixtures with solutes such as small molecules (e.g., ethanol,^19^ dimethyl sulfoxide,^20^ acetone,^20^ and methane^21^) as well as larger molecules and aggregates such as pentanol,^16^ hexadecane,^22^ surfactant micelles,^23,24^ and polymers.^25,26^ For instance, Raman MCR has revealed that near ambient temperature, hydration shells of small molecules such as methane and *n*-butanol possess a hydrophobically enhanced water structure with greater tetrahedral order and fewer weak hydrogen bonds than the surrounding bulk water.^16,21^ It has also been observed that oil–water droplet interfaces can exhibit reduced tetrahedral order and weaker water hydrogen bonding, along with a substantial population of dangling hydroxyl groups.^22^

While revealing, Raman MCR studies have been largely focused on low concentrations of single solutes. Ternary solvents, however, can exhibit molecular and macroscale properties that are distinct from those of the binary mixtures of their constituents; e.g., the effects are nonadditive, and the ternary mixture properties would not be intuited from linear combinations of the binary mixture properties. For example, some ternary mixtures consist of water, a water-immiscible oil, and a cosolvent miscible with both oil and water. This three-component mixture can form heterogeneous nanoscale solvent domains^27,28^ for compositions that fall within the macroscopically single-phase region of the ternary phase diagram. Ternary solvent organization into oil-rich or water-rich aggregates has been called by a variety of names in the literature, including nanophases (our preferred terminology),^29^ nanoscale heterogeneities,^30^ surfactant free microemulsions,^31,32^ mesoscale structures,^33^ and pre-ouzo structures.^34^ Notably, neither the oil nor cosolvent is sufficiently amphiphilic to self-assemble independently in water (i.e., does not form micelles or vesicles), yet there is an emergence of nanoscale solvent organization within the ternary mixture. The general explanation for this nanophase organization, which is believed to be thermodynamically stable, is that the hydrophobic nature of the oil drives aggregation, which is balanced by hydration forces and entropy.^35^ Strong noncovalent interactions, such as hydrogen bonding, facilitate the formation of nanophases.^29^ The presence of nanophases in ternary mixtures has been confirmed by a variety of analytical techniques, including conductivity,^36^ UV–Vis with solvatochromic probes,^31^ dynamic light scattering (DLS),^37,38^ cyclic voltammetry,^39^ small-angle neutron scattering,^40^ small-angle X-ray scattering,^35^ etc. The presence of these nanophases can impact chemical kinetics and reactivity.^41–46^ For instance, oil-in-water nanophases can induce faster azide–alkyne click reactions between hydrophobic reagents by facilitating reagent colocalization,^46^ while water-in-oil nanophases can preserve enzyme activity and prevent denaturation in otherwise inhospitable solvents.^42^ Recent studies have also highlighted a growing interest in using nanophases as reaction media for polymer synthesis and nanoparticle fabrication.^17^

In this work, we use Raman MCR to investigate changes in the structure of water as a function of the ternary solvent composition. Specifically, we use ternary mixtures of water, a cosolvent (acetone, dimethylformamide, or dimethyl sulfoxide), and oil (2-hexanone or 2-pentanone), chosen such that the O–H vibrational signal from water does not overlap with any other vibrational resonances in the mixture. To maintain a constant water molarity, the oil and cosolvent concentrations were varied reciprocally. Hence, we can directly compare the amounts of perturbed water as well as probe differences in the distribution of hydrogen-bonding strengths as a function of ternary composition using Raman MCR. Our experimental results reveal that the addition of oil at the expense of the cosolvent in a ternary mixture leads to a decrease in total Raman MCR area, signifying reduced apparent extent of solute-correlated water perturbation. However, more dangling hydroxyl groups are formed as oil is added, suggesting the formation of an increased hydrophobic–water interface. Throughout this work, the Raman MCR area is interpreted as an apparent spectroscopic measure of the extent of solute-correlated water perturbation rather than a direct measure of the volume of a discrete hydration shell, particularly in these concentrated multicomponent mixtures. Taken together, we propose that these observations suggest that oil incorporates into nanoscopic oil-in-water domains, perhaps with cosolvent enriched at the interface. These nanophase domains can be detected by DLS for some ternary compositions. Such findings provide a deeper understanding of how hydrophobic hydration influences nanophase formation in ternary solvent systems, offering new insights into solvent behavior and water structure in complex mixtures.

## Results

We used Raman MCR to characterize the organization of water in ternary solvents composed of water, a water-immiscible oil, and a cosolvent miscible with both oil and water. Specifically, we selected the cosolvents acetone, dimethylformamide (DMF), and dimethyl sulfoxide (DMSO), and the oils 2-hexanone or 2-pentanone (Figure 1a). The various combinations of these liquids have different strengths and types of intermolecular interactions, including London dispersion, dipole–dipole, dipole–induced dipole, *n*-Π*, and hydrogen bonding interactions. The ternary phase diagrams for all combinations of solvents have both a macroscopically homogeneous single-phase region as well as a heterogeneous two-phase region (Figure 1b). Notably, alcohols and carboxylic acids were avoided due to spectral overlap among water, alcohol, and carboxylic acid’s O–H stretching band at 3000–3600 cm^–1^, which would make it difficult to isolate the Raman response from just the water. The oils and cosolvents that were selected have no spectral overlap with the O–H of water.

**Figure 1:**
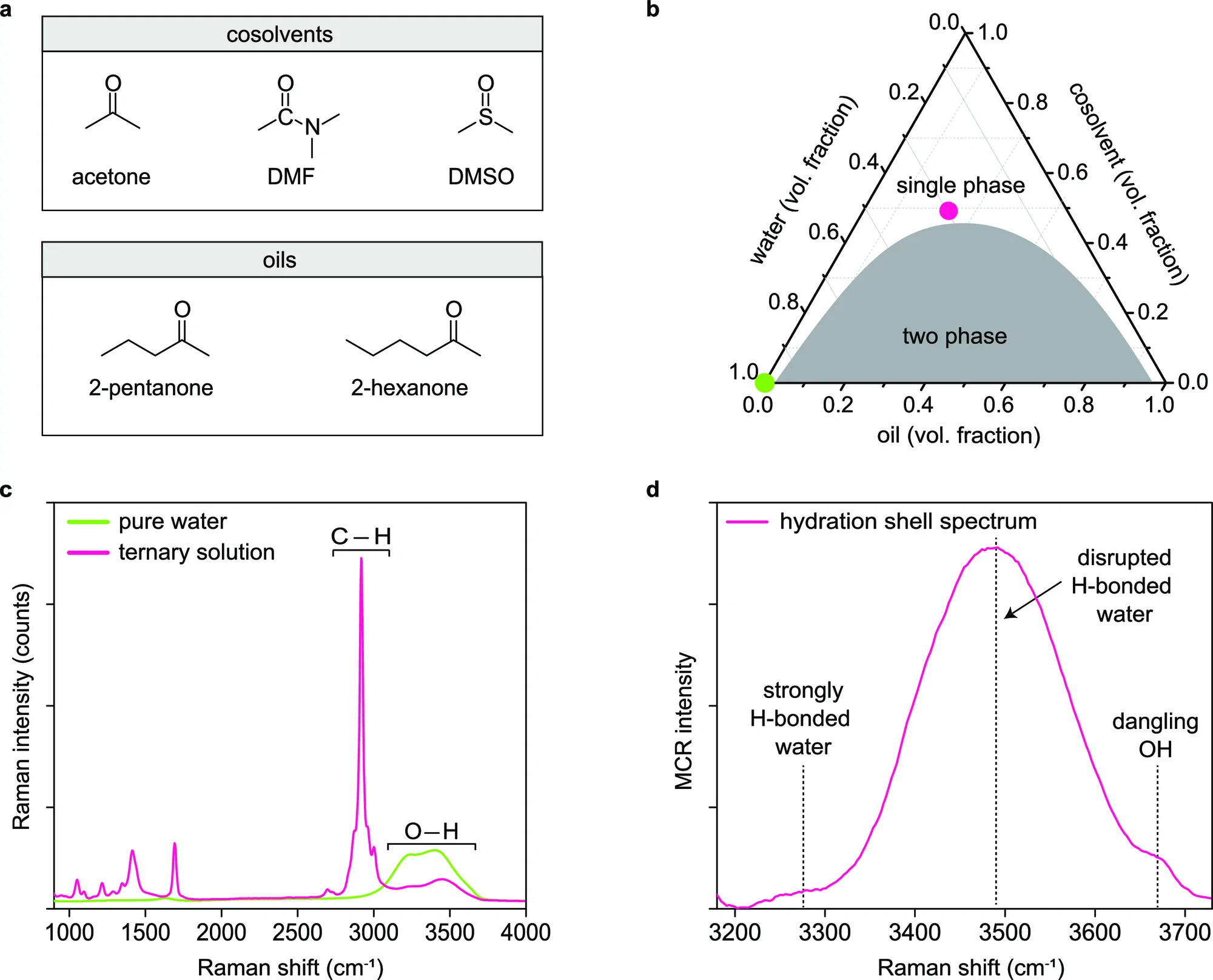
Raman MCR spectra can be used to investigate water structure in a ternary mixture of oil, water, and cosolvent. (A) Chemical structures of the molecules employed as oils and cosolvents in this work. (B) General schematic of a ternary phase diagram of oil, water, and cosolvent with two-phase and single-phase regions. The green and pink dots represent illustrative compositions, analyzed by Raman spectroscopy, with corresponding color-coded spectra shown in **C**. (C) Exemplary Raman spectra of pure water (green line) and a ternary solution (pink line). (D) Solute correlated Raman MCR spectrum of the solution, which provides information about the structure of water in the hydrophobic hydration shell.

The general process followed for Raman MCR analysis is given in Figure 1. The Raman spectrum of pure water was first collected, which exhibits a broad band in the O–H stretching region (green spectrum in Figure 1c), centered at ∼3450 cm^–1^. Water’s O–H stretching frequency is correlated with hydrogen bond strength: strengthening of the hydrogen bond weakens the parent O–H bond, resulting in a red shift of the stretching frequency. Conversely, weakening the hydrogen bond shifts the O–H stretching frequency toward higher wavenumbers (blue shift). Changes to water’s bonding and organization caused by the addition of solutes (pink spectrum) can be seen by directly comparing the mixture’s spectrum with the pure water spectrum (Figure 1c). The measured Raman spectrum of the solution (*S*_total_) is a linear combination of the water solvent spectrum (*S*_1_) and the solute correlated spectrum (*S*_2,_ the MCR spectrum).^18,47,48^ *S*_2_ includes contributions from the solutes themselves (oil and cosolvent) as well as water molecules whose vibrational modes are perturbed by the solute due to physical proximity. To calculate *S*_2_, we took the non-negative minimum area difference between *S*_total_ and *S*_1_, which removes the contribution from bulk (unperturbed) water and enables analysis of the water within the solute’s hydration shell (Figure 1d). The area under the MCR curve correlates to the amount of perturbed water in the hydration shell of solute molecules. The broad O–H stretch of water in the MCR spectrum has three different spectral components that represent water molecules with varying hydrogen bond strengths (Figure 1d):^49–51^ the low frequency band (∼3250 cm^–1^) was attributed to strongly hydrogen bonded water, mid frequency (∼3480 cm^–1^) was assigned to more disrupted hydrogen bonding, and the high frequency resonance (∼3670 cm^–1^) corresponds to either very weakly hydrogen bonded water^52^ or dangling hydroxyl groups.^19^

The protocol described in Figure 1 for Raman MCR was used to investigate hydrophobic hydration (Figure 2). The impact on the water structure by both the cosolvent (DMF, DMSO, or acetone) and the oil was explored. We used either 2-hexanone or 2-pentanone as the oil (Figure 1a),^29^ the choice of which was dictated by the cosolvent and the resulting area of the single-phase region in the ternary phase diagram. 2-Hexanone/DMSO/water contained too small of a single-phase region (Figure S1), so we used the less hydrophobic oil, 2-pentanone, while 2-hexanone was used with acetone and DMF. For each solvent system, we collected Raman MCR spectra for a series of compositions within the single-phase region of the phase diagrams that all have the same molarity of water, 17.1 ± 0.1 M (Figure 2a**-**i, b**-i**, c**-i**). Importantly, the experiments were designed such that the MCR spectral intensities and fitted areas for each ternary composition should be directly comparable (Figure 2a-ii, b**-ii**, c**-ii**). The precise molar compositions for each mixture are in Table S1.

**Figure 2:**
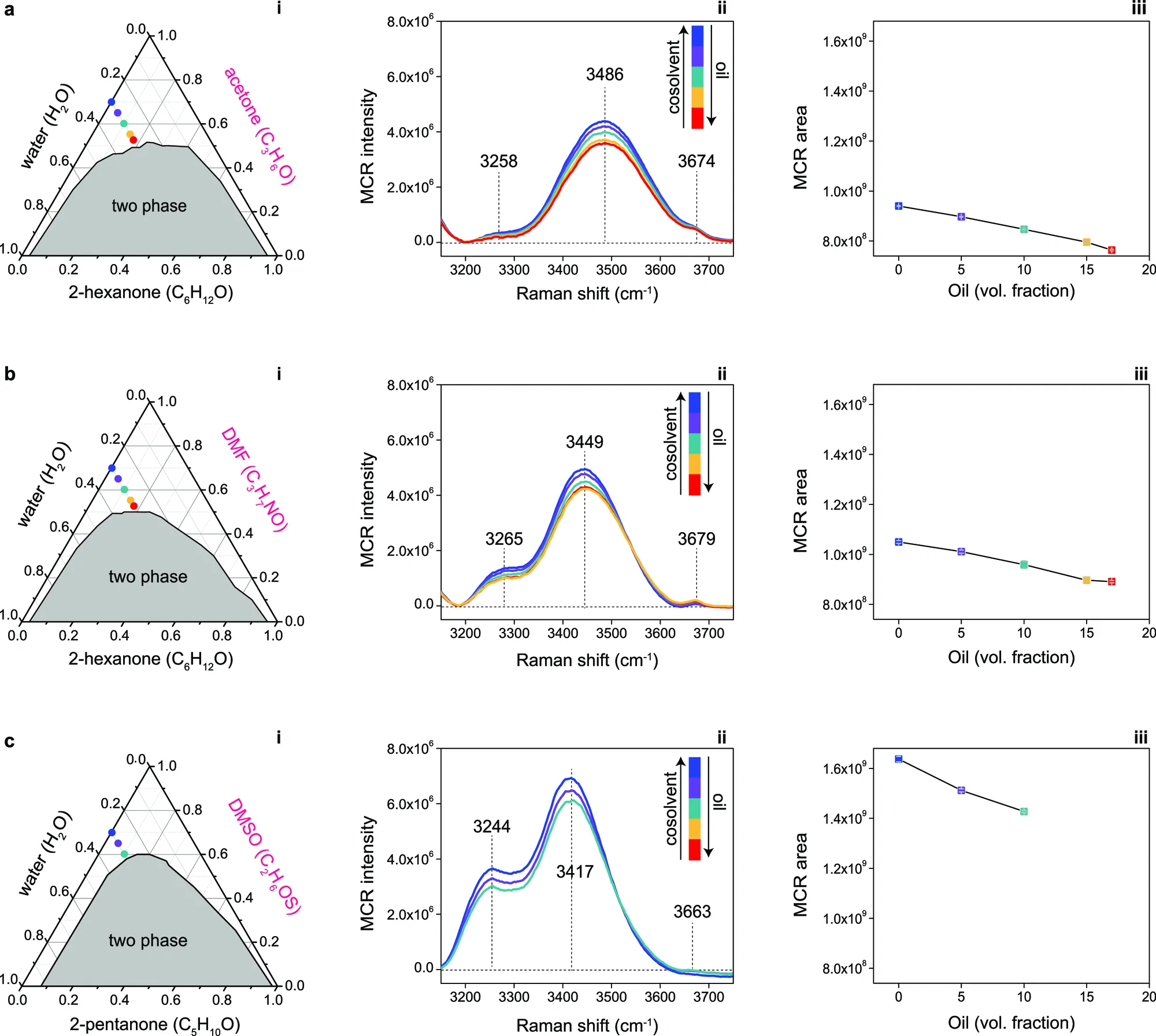
Raman MCR analysis illustrating the trend in hydrophobic hydration as a result of the gradual addition of oil at the expense of cosolvent. (A, B, C-i) Ternary phase diagrams of acetone/water/2-hexanone, DMF/water/2-hexanone and DMSO/water/2-pentanone with colored dots corresponding to the specific compositions analyzed by Raman MCR. (A, B, C-ii) Raman MCR spectra of compositions marked in the ternary phase diagrams, color-coded. (A, B, C-iii) Variation in total MCR area from the spectra in (**-ii**) plotted as a function of oil volume fraction. (Raw data in Table S2). In all three systems, the MCR area decreased with the addition of oil. Each composition was measured three times, and one spectrum is plotted as an example in (ii); the spectra were highly reproducible. The MCR area for each spectrum was calculated, and the average and standard deviation are shown in (iii). The raw Raman MCR spectra are provided as Supporting Information files.

For binary water-cosolvent compositions with no oil (blue dots on the phase diagrams in Figure 2), the peaks in the MCR spectra were blue-shifted compared to the Raman spectrum of pure water (Figure S2, **light blue line**).^53,54^ To make sure this trend was not due to a change in the bulk properties of water, since we were adding high concentrations of cosolvent (∼10 M), we also conducted Raman MCR analysis at lower cosolvent concentrations (2.7 M) and still observed the same trend (Figure S2). The shift was most pronounced with acetone and DMF (81 cm^–1^ and 41 cm^–1^ for the high frequency band, 15 cm^–1^ and 22 cm^–1^ for the low frequency band, respectively), while almost negligible for DMSO. This blue shift of the water O–H stretch in the acetone system indicates that water is more weakly bonded in the hydration shell of acetone compared to DMF and DMSO; this observation is consistent with prior reports.^20^ The shoulder peak around 3250 cm^–1^ decreased for all three cosolvents in water and almost completely disappeared in the SC spectrum of the acetone/water system (Figure S2a), consistent with a decrease in the population of strong hydrogen bonds. The area under the weakly bonded/dangling O–H peak at ∼3670 cm^–1^ was also substantially higher for the acetone/water mixture (Figure S2c).

As oil was added to binary mixtures to create ternary solutions, we found that the total MCR area within 3100–3700 cm^–1^ decreased in all three systems (Figure 2a,b,c**-ii** and a,b,c**-iii**, Table S2). Note that oil was added at the expense of the cosolvent to maintain a constant molar concentration of water for all compositions. This gradual decrease in MCR area was more pronounced in the case of DMSO/2-pentanone/water, as indicated by the slope of the MCR area vs oil volume fraction plots in Figure 2c-iii. Since oil is more hydrophobic than the cosolvent, one might expect that introducing more oil would perturb more water; for instance, it has been shown previously that hexadecane perturbs more water than hexane on a molar basis.^24^ However, the fact that we observed a *decrease* in the total MCR area indicates that the added oil did not interact with the bulk water to the extent that might be anticipated.

To gain greater insight into the hydration shell structure, we used Gaussian fitting to isolate and quantify the area under the three O–H stretch peaks, each of which represents a different hydrogen bonding strength (Figure 3a, Table S3). The peak area ratio A_3250/3450_ was used as a metric for the degree of water “structuring”,^55^ whereby a higher value corresponds to increased water ordering. As can be seen in Figure 3b, A_3250/3450_ either remained relatively constant or decreased with the addition of oil, implying a disruption of the tetrahedral structure of water at higher oil concentrations. The variation in the area of dangling O–H peak at ∼3670 cm^–1^ as a function of oil concentration was particularly interesting: addition of oil caused an increase in the dangling O–H for all systems (Figure 3c) despite an overall decrease in the total MCR area (i.e., total perturbed water) (Figure 2a,b, c-ii). Acetone-containing ternary solvents formed the highest number of dangling O–H bonds, while DMSO had the fewest.

**Figure 3:**
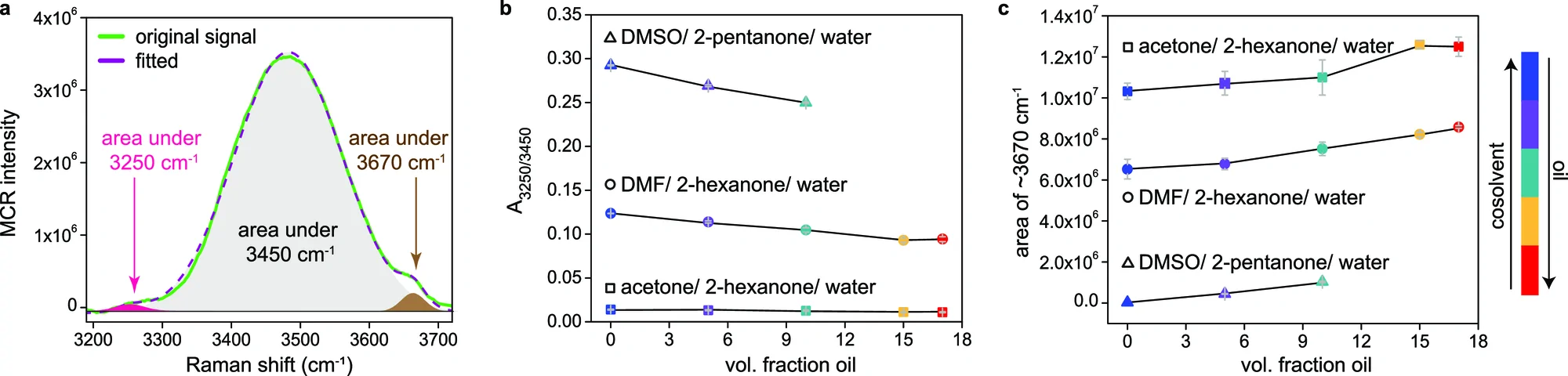
Variation in the structure of water in the hydration shell for three different ternary solvent systems. (A) Exemplary MCR spectrum of Gaussian fitting to three peaks, each representing different strengths of hydrogen bonding. Refer to Figure 1D for peak descriptions. (B) The area ratio of two peaks, A_3250/3430_, versus oil volume fraction; this peak ratio is an indicator of the degree of water ordering, where higher values correlate to greater water structuring (more tetrahedral water). (C) A gradual increase in the area of the peak representing dangling O–H is observed upon oil addition. Table S3 contains the raw data.

We aimed to investigate how the hydrophobic hydration trends correlate with the physical properties of solution and nanophase formation measured by another method: DLS. Nanophases have been characterized by a variety of methods, but we chose DLS because it is an established and readily accessible method to analyze the presence of scattering centers in ternary solutions, a positive indicator of nanophase formation.^29,38,56^ In DLS, scattering nanophases undergo Brownian motion, producing time-dependent fluctuations in the scattered light intensity that are used to construct an autocorrelation function (ACF). The ACF reveals the presence of scattering species through the signal strength and provides estimates of their size distribution based on characteristic decay times. Higher correlation coefficients at short delay times indicate more well-defined and concentrated scattering species. While DLS offers a rapid and accessible means for screening solvent structuring, we note that it has limitations, including an inability to detect very small (<1–2 nm), dilute (or very concentrated), or rapidly fluctuating clusters, which would require techniques such as neutron scattering to detect. We analyzed selected compositions by DLS, and the corresponding ACFs are shown in Figure 4.

**Figure 4:**
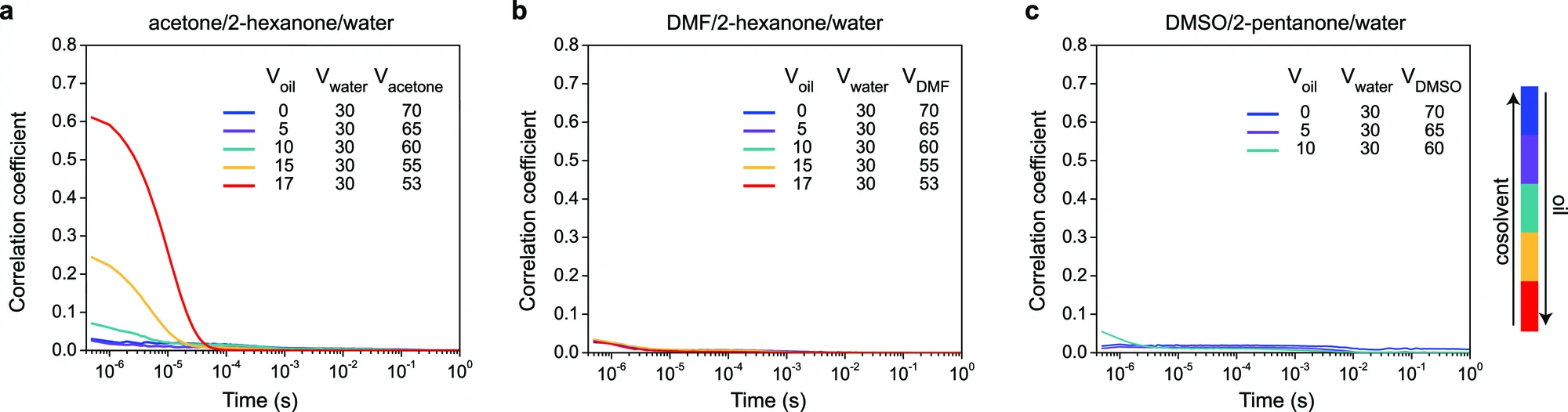
DLS analysis of ternary solutions reveals scattering from nanophases. DLS analysis of selected compositions of ternary solvents, colored lines correspond to volume fraction of oil, water, and cosolvent. The ACF reveals strong scattering from nanophases in the case of 2-hexanone/acetone/water (a), while less detectable scattering in the case of 2-hexanone/DMF/water (b) and 2-pentanone/DMSO/water system (c), suggesting that if the solvent aggregates indicated by Raman MCR are present, they fall below the detection limit of DLS.

The most scattering (highest ACF intercept) was observed with the acetone/2-hexanone/water system; interestingly, this was also the system that generated the most dangling O–H, suggestive of more well-defined oil–water interfaces compared to the other mixtures. We estimated the hydrodynamic diameter of nanophases in exemplary acetone-containing ternary solvents to be approximately 3.6–3.7 nm, a value which is in line with prior studies of nanophase dimensions (but in differing chemical compositions) by analysis methods such as DLS,^38^ SAXS,^57,58^ and neutron scattering.^40^ For DMF/2-hexanone/water and DMSO/2-pentanone/water, minimal scattering was observed in DLS after the addition of oil. This result admits two interpretations. First, oil-rich nanodomains may still form in these systems but at sizes too small (roughly <1–2 nm) or with too little scattering contrast to be resolved by light scattering under our experimental conditions. Second, oil in the DMF and DMSO systems may instead be solvated more effectively at the molecular level by the cosolvent, rather than forming discrete oil-rich domains as in the acetone system.

## Discussion

Two key takeaways from the Raman MCR analysis are that introducing oil at the expense of cosolvent in the ternary mixture leads to (1) *less* total perturbed water but (2) *more* dangling O–H. This effect becomes more pronounced at higher oil concentrations. Because oil is added at the expense of the cosolvent in these ternary mixtures, the observed decrease in MCR area could in principle arise from the removal of a strongly water-interacting cosolvent rather than from oil aggregation alone. A full additive model, incorporating the binary water/cosolvent and water/oil contributions, is not feasible for these systems because 2-hexanone and 2-pentanone have extremely limited aqueous solubility; the resulting binary water/oil mixtures yield Raman spectra with a very weak signal, precluding reliable MCR analysis of this component. To isolate the effect of oil addition from that of cosolvent dilution, we instead compared the MCR area of the binary water/cosolvent mixture to that of the ternary mixture at the same cosolvent concentration (Figure S3); the ternary mixture had a lower MCR intensity. Because the cosolvent concentration is held constant between these comparisons, the observed decrease in the MCR area upon oil addition cannot be explained by cosolvent dilution alone. This suggests that the reduction reflects a perturbation of the water hydrogen-bonding network arising from the combined interactions among the water, cosolvent, and oil. Our explanation for these trends is shown in Figure 5. We postulate that these results indicate that added oil preferentially interacts with the cosolvent (and other oil molecules) to form oil- and cosolvent-rich aggregates dispersed in water, with the cosolvent likely enriched at the water interface of the aggregate. Since cosolvent and oil that partition into the aggregates would be less solvated by water, there would be a reduction in total perturbed water as a function of oil addition.

**Figure 5:**
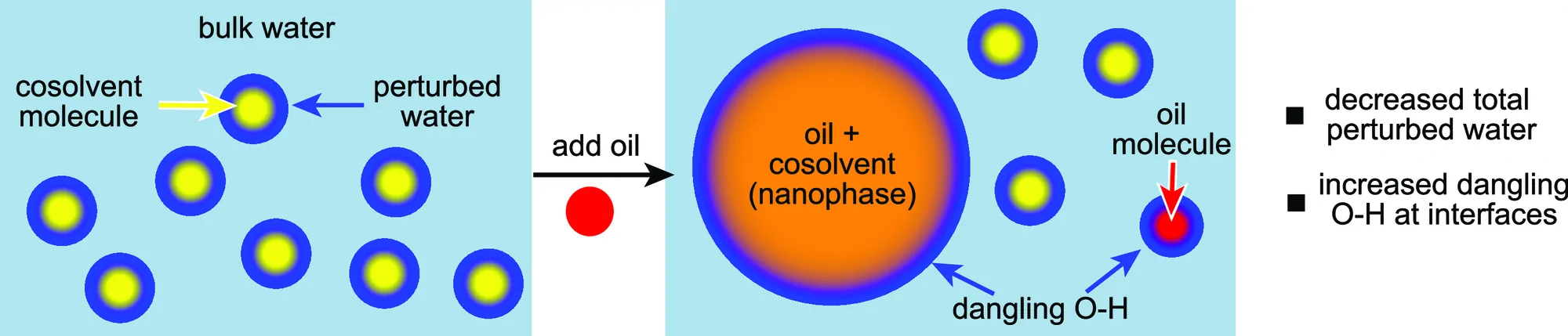
Schematic of the hydration shell of cosolvent molecules and the effect of added oil on the water structure at the oil–water interface in a ternary solvent system. Left schematic: the binary mixture of bulk water (light blue), cosolvent (yellow), and perturbed water (dark blue). Right schematic: when oil (red) is introduced to a binary mixture, oil and cosolvent-rich nanophases form. Cosolvent partitions from the bulk into the oil-rich nanophase. Oil is also sequestered from water, forming an oil–water interface. This organization leads to an overall decrease in total perturbed water and increases the population of dangling O–H bonds.

Cosolvents are more likely to perturb water on their hydrophobic side (e.g., the methyl group) as opposed to the carbonyl side; to the extent that the methyl groups can face toward the inside of the aggregate, this would reduce total solute correlated water perturbation as well. The increased dangling OH could be from two sources: either molecularly dissolved oil in water, or from hydrophobic chains protruding into the water from within a nanophase. All three ternary mixtures examined by Raman MCR exhibited these same trends, although notably, the acetone mixtures had the lowest total MCR area but also the highest number of dangling OH groups. Nanophases in the acetone mixtures were detectable by DLS, as indicated by strong scattering; however, the lack of scattering from the DMF and DMSO mixtures does not rule out the existence of nanophases, but may indicate that if nanophases are present, they are either too highly fluctuating or too small to be detected by DLS. Previous studies have examined ternary systems of DMSO/water/oil and DMF/water/oil and found indications of nanophases, particularly in cases where the oils have stronger intermolecular interactions with the water and cosolvent (e.g., alcohols, which we avoided in this study).^59–61^ Binary mixtures of DMSO/water and DMF/water have also been observed to exhibit molecular-scale heterogeneities by methods like Fourier transform infrared (FT-IR) spectroscopy, and ultrafast IR spectroscopy,^62,63^ X-ray diffraction,^64^ and supported by molecular dynamics simulations.^65,66^ Hence, we believe that the Raman MCR results yield insight into the formation of heterogeneous solvent organization as a function of ternary solvent composition, which is not evident from DLS. Alternatively, oil in the DMF and DMSO systems may be more effectively solvated at the molecular level by the cosolvent, rather than forming discrete oil-rich domains as in the acetone system. This is consistent with the small-to-negligible change in dangling O–H intensity we observe for the DMF and DMSO systems relative to acetone; if oil were forming water-excluding domains comparable to those in the acetone system, we might expect a similarly pronounced increase in dangling O–H signal, which we do not observe.

Beyond the trends in the MCR peak areas as a function of oil concentration, the peak positions are also informative. Although all systems have a 3250 cm^–1^ peak associated with the most rigid (ice-like) structure of water, this peak is reduced by the gradual addition of oil in ternary solvent systems, as can be seen by Raman MCR; the acetone mixtures in particular have a very small 3250 cm^–1^ peak. We also found that the MCR spectrum of the acetone-based ternary system is blue-shifted relative to the DMF- and DMSO-based systems, indicating a comparatively weaker hydrogen-bond network in the hydration shell. Our experiments also show a gradual increase in the intensity of the higher frequency peak (∼3660 cm^–1^), a peak that is usually associated with dangling OH groups around dissolved nonpolar groups, as a function of oil concentration for all systems. However, introducing more oil molecules to water/cosolvent mixtures does not significantly shift the dangling O–H stretching frequencies. In fact, no change was observed in the acetone or DMSO mixtures, and for DMF, a small redshift of 9 cm^–1^ was found between the binary mixture and that which had the highest oil volume. Prior reports suggest that the dangling OH peak at the interface of hexadecane oil-in-water drops is red-shifted by 95 cm^–1^ compared to a flat oil interface due to a strong electric field.^22^ We do not observe such a significant shift here, which may be consistent with the more transient nature of the hydrophobic nanophases that do not have as clearly defined of an oil–water interface. Although distinct from critical fluctuations,^67,68^ nanophases are highly dynamic and constantly fluctuating, so their interfaces are not expected to be as well-defined as the interface of oil-in-water droplets.

All examined compositions in this work fall within the single-phase region of the ternary phase diagram, so we anticipate that as more oil is added, macroscopic phase separation would yield the formation of much larger, coalescing oil droplets that would have properties comparable to prior Raman MCR analysis of droplet interfaces. This work thus yields insight into how the nanoscopic processes, properties of the water, and organization of molecules in ternary mixtures evolve as a function of composition en route to macroscopic phase separation. Understanding these solvent properties is important for exploiting ternary single-phase mixtures as solvents for reactions, catalysis, or materials synthesis.

## Conclusion

In this work, we investigated how oil solubilization into water/cosolvent mixtures affects the structure and hydrogen-bonding network of water. Using Raman spectroscopy combined with multivariate curve resolution (MCR), we characterized water structure in three systems: acetone/2-hexanone/water, DMF/2-hexanone/water, and DMSO/2-pentanone/water. Across all ternary mixtures, we observed a pronounced decrease in the MCR area under the curve in the OH stretch region upon the addition of oil at the expense of the cosolvent, while maintaining a constant concentration of water. This reduction indicates that the total population of perturbed water molecules in the hydrophobic hydration shells decreases as oil is added. This result is counterintuitive and suggests that added oil does not perturb as much water as might be otherwise anticipated. In addition, the population of dangling O–H groups increases upon oil addition in all mixtures, with the most significant population found for the acetone-based system; this was also the mixture that exhibited the strongest scattering in DLS. The emerging picture is that oil-rich nanophases in water form upon oil addition to binary water/cosolvent mixtures, which reduces the total perturbed water despite the formation of mixtures that have a greater degree of hydrophobicity. However, we do not observe significant Stark shifts upon oil addition, which may suggest that any oil–water interfaces present are not as well-defined or stable as the interfaces of oil-in-water droplets that would be formed from ternary compositions falling within the two-phase region of the phase diagram. Overall, these findings demonstrate that cosolvent molecular structure plays a measurable role in determining water organization in complex ternary solvent systems. Moreover, the introduction of oil to create a ternary solvent leads to heterogeneous solvent organization. Our results provide insight into molecular distribution and interfacial restructuring in multicomponent mixtures, with broader implications for understanding solvation, emulsification, and interfacial phenomena in soft matter systems and complex solutions.

## Supplementary Material





## Notes

Materials and methods, supporting figures and supporting tables (PDF)
MCR spectra of solutions (XLSX)
