# Supplementary material for: Water Structure within Ternary Solvents Suggests Nanophase Formation
Source: J Am Chem Soc. 2026 Aug 17;148(33):36044–52. doi: 10.1021/jacs.6c10066 (PMC13523747; doi:10.1021/jacs.6c10066)
Supplement: Supplementary file 1 [file ja6c10066_si_002.pdf]

Supporting information

Water structure within ternary solvents suggests nanophase formation

Binish Ashfaq[1], Chun-Ting Lin[1], Paul S. Cremer[1,2], Lauren D. Zarzar[1,3,4*]

1. Department of Chemistry, The Pennsylvania State University, University Park, PA 16802, USA
2. Department of Biochemistry and Molecular Biology, The Pennsylvania State University, University Park, PA 16802, USA
3. Department of Materials Science and Engineering, The Pennsylvania State University, University Park, PA 16802, USA
4. Materials Research Institute, The Pennsylvania State University, University Park, PA 16802, USA

*Corresponding author, ldz4@psu.edu

This document contains

1. Materials and Methods
2. Supporting Figures
3. Supporting Tables
4. References

1. Materials and Methods

Materials

Acetone (~99.5%) (VWR Chemicals); N, N-dimethylformamide (DMF) (>99.8%) (Macron Fine Chemicals); Dimethyl sulfoxide (99.9%) (EMD Millipore Corporation), 2-hexanone (>98%) (Toyoko Chemical Industry); 2-pentanone (99%) (Beantown Chemical); deionized (DI) water. All pure solvents were filtered before use by employing different syringe filters based on the compatibility of solvents with the filters. DI water was filtered by nylon filters (0.1 μm), and all other solvents were filtered with PTFE syringe filters (0.22 μm).

Building ternary phase diagrams

Ternary phase diagrams were determined at ~22 °C using volume measurements. Oil–water mixtures (1 mL, 5 vol% intervals) were titrated with cosolvent until a clear, single-phase formed. The corresponding component volumes were recorded. Single-phase regions appeared transparent, while a visually heterogeneous mixture was defined as multiple phases. By repeating the above-mentioned procedure, the boundary between single phase and multiple phase was determined.

Measurement of the molarity of components in the ternary mixture

Moles of each component in a mixture were calculated by using the density and molecular weight of pure solvent ($n = density * added\ volume/molecular\ weight$). The mass of the mixed solvent was measured by using a high performance precision balance by OHAUS Scout™. The mixture density was recorded at 22 °C using an Anton Paar DMA 1001 vibrating U-tube densitometer, calibrated with ultrapure water (0.99799 g/cm³ ± 0.00002). Measurements were taken in precision mode. The mixture density and mass enabled the calculation of the final mixture volume. Knowing the number of moles of each component solute and the total mixture volume enabled calculation of the molarity of each component. Between samples, the cell was rinsed three times with acetone and dried with continuous air flushing until readings stabilized. Reported weights and densities are listed in **Table S1**.

Raman spectroscopy

A home-built Raman spectrometer was used to acquire all Raman spectra. Excitation was provided by a 640 nm 100 mW laser (CrystaLaser, Reno, NV, USA). The power and polarization of the laser line were controlled by two half-wave plates (1200-15; CVI Laser, Albuquerque, NM) positioned on either side of a beam splitter (CCM1-PDBS252; Thor Labs, Newton, NJ). Highly reflective mirrors (BB1-E02P; ThorLabs) and a 638 nm ultrasteep long-pass filter (LP02-638RE-25; Semrock) directed the beam to the sample.

The laser was focused onto a 1 cm quartz cuvette (5061-3387; Agilent Technologies) housed in a temperature-controlled jacket (Versa 20; Quantum Northwest, Liberty Lake, WA) using a 10× objective (LU Plan, WD = 17.3 mm, NA = 0.3; Nikon, Japan). Backscattered, unpolarized Raman signals were collected and passed through two 638 nm Ultrasteep long-pass filters before being focused by a lens (AC254-050-A; Thor Labs) onto a custom 1 × 15 fiber-optic bundle (FiberTech-RoMack, Williamsburg, VA) coupled to a spectrometer (IsoPlane SCT 320; Princeton Instruments, Trenton, NJ). A 300 g/mm grating (unless otherwise noted) dispersed the light onto a liquid-nitrogen-cooled 100 × 34 CCD array detector (Pylon-100BR; Princeton Instruments).

The power of the incident light at the sample stage was adjusted to 50 mW for all spectra. The exposure time for the CCD camera was adjusted to ensure that the signal did not saturate the detector (0.01 s), and the number of exposures was 2400 per frame. The spectrum of each solution was measured in duplicate. Measurements were performed at 22 °C. Hydration shell analysis was benchmarked to a spectrum of pure DI water that was collected right before the solution spectrum.

Multivariate Curve Resolution (MCR) analysis

The hydration shell spectra were obtained using a multivariate curve resolution (MCR) algorithm written for IGOR Pro (Wavemetrics Inc., Portland, OR). This MCR code was generously provided by Prof. Dor Ben-Amotz at Purdue University (West Lafayette, IN)[1]. The inputs for the MCR analysis were a spectrum of solvent mixture (S_{total}) and a spectrum of pure deionized water (S_1). The output was the deconvolution of the total solution spectrum into components from pure DI water and another from the solute-correlated spectrum (S_2), as described by the equation below. The magnitude of each component was quantified by the two positive coefficients c_1 and c_2.

$$S_{total} = c_1 S_1 + c_2 S_2$$

The hydration shell spectrum was defined by the mathematical solution to Equation above that minimized the value of c_2. As such, the hydration shell spectrum represents the minimum area difference between the spectra of the solvent mixture and pure DI water. The convergence of the MCR analysis to this condition was confirmed when the solute-correlated spectrum was minimized relative to the background signal, not necessarily zero. MCR analysis was performed over a frequency range from 2360 to 4145 cm^{-1}.

Dynamic Light Scattering (DLS) analysis of ternary solvent systems
Selected ternary samples were analyzed by DLS at 25 °C by using a ZEN 3600 Zetasizer Nano ZS, Malvern Instruments. The instrument is equipped with a He-Ne laser (632 nm) as the light source and a temperature control accessory. All pure solvents were filtered with a 0.22 µm PTFE syringe filter, while DI water was filtered by nylon filters (0.1 µm). All binary and ternary solvent systems were prepared by mixing the already-filtered pure solvents, and then the mixture was filtered again using PTFE filters (0.22 µm). 1 mL of sample solution was loaded in a glass cuvette having a 1 cm path length, and each sample was allowed to equilibrate for 60 seconds before running the experiment. DLS analysis was repeated three times for each sample.

Hydrodynamic diameter determination. The hydrodynamic diameter of selected composition from acetone/2-hexanone/water system was calculated from the autocorrelation function using the Stokes–Einstein equation. This requires the diffusion coefficient (D), obtained by fitting the correlation curve to an exponential decay, with D proportional to the decay lifetime.

$$D = {k_B T}/{6\pi\eta R_H}$$

where k_B is the Boltzmann constant (1.380 × 10^{-23} kg m^2 s^{-2} K^{-1}), T is the absolute temperature, and η is the solution viscosity. Because the viscosity of the ternary mixture deviates from that of the pure solvents, the viscosity of selected samples was measured experimentally, as described below.

Viscosity measurement
Viscosities of the ternary mixtures were determined at room temperature (~22 °C) using a Cannon–Fenske viscometer (cell constant = 0.003570 cSt/s). Between measurements, the viscometer was rinsed three times with acetone and dried under compressed air. For each composition, 7 mL of sample was loaded into the viscometer and drawn to the upper fiducial mark under vacuum, and the efflux time between the two calibration marks was recorded in triplicate. Kinematic viscosity (cSt) was calculated as the product of the average efflux time and the cell constant, and the dynamic viscosity (cP) was obtained by multiplying the kinematic viscosity by the solution density (g/mL), measured as described above. All measured viscosities are reported in **Table S4**.

2. Supporting figures

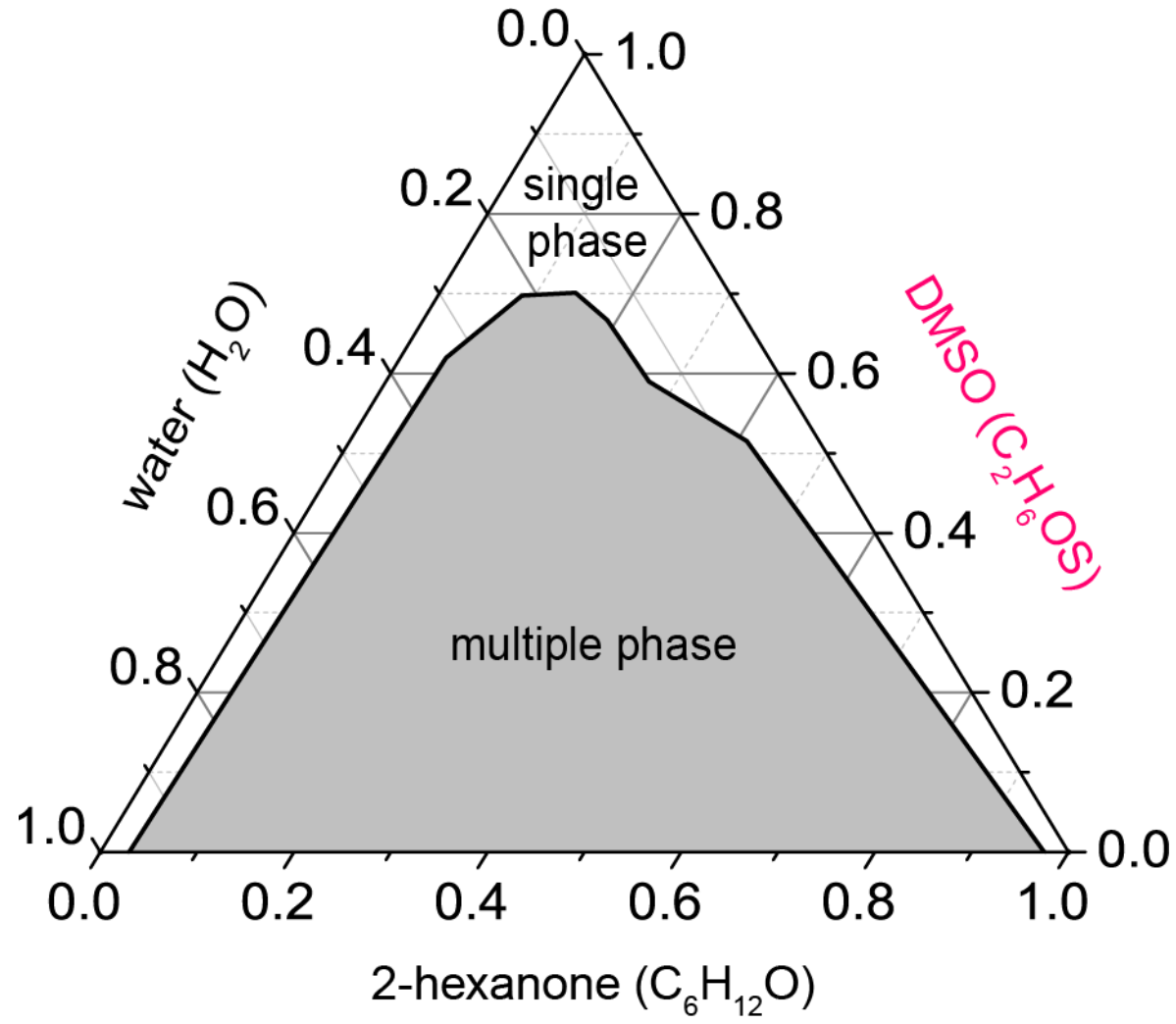

Figure S1. Ternary phase diagram of 2-hexanone/DMSO/water.

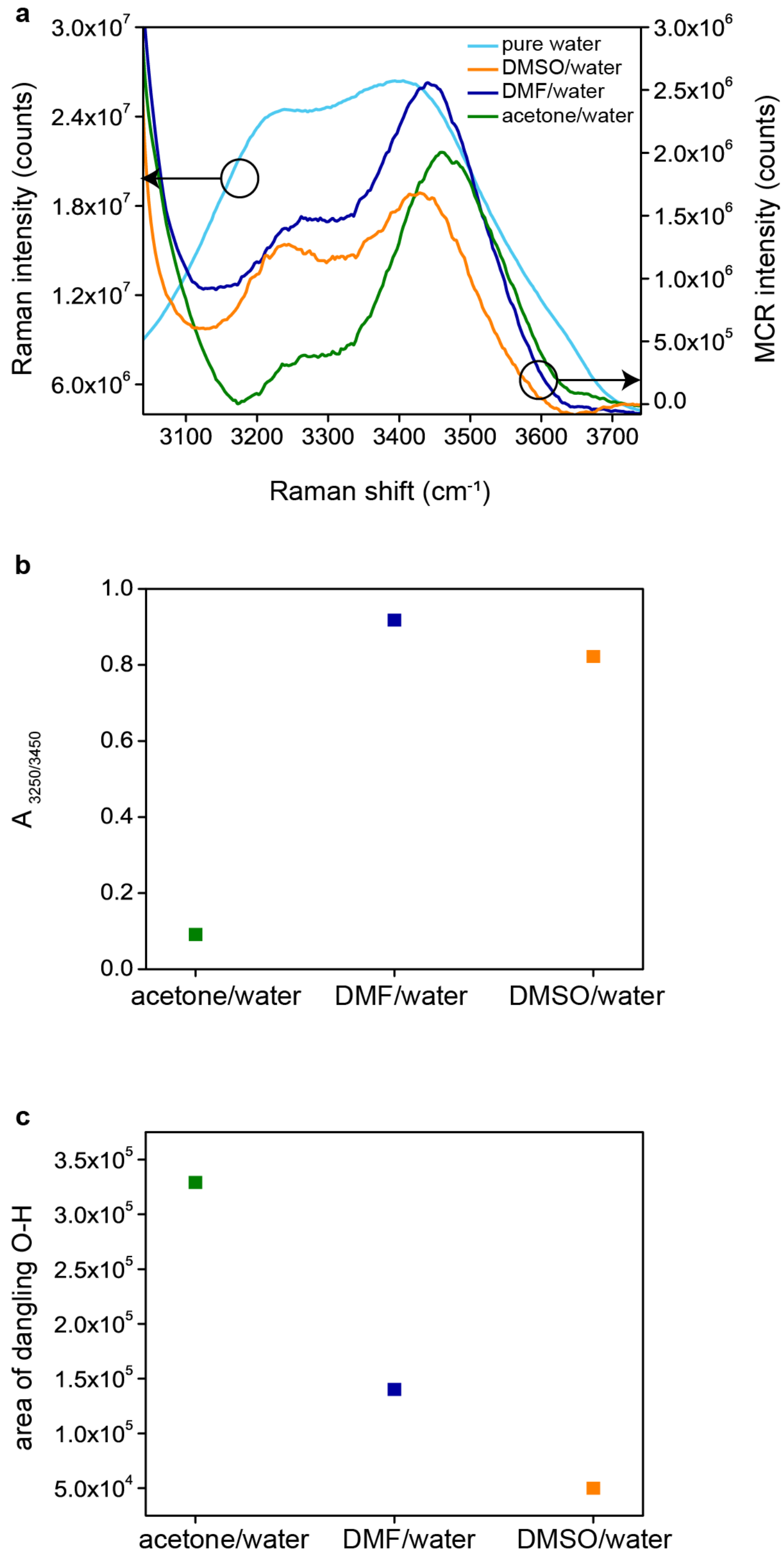

Figure S2. Raman MCR analysis of binary mixtures of water and cosolvent. A) Raman spectra of pure water (light blue line) versus MCR spectra of three different cosolvents (2.7 M in water). **B)** The area ratio of two peaks (~3250 cm^{-1} / ~3400 cm^{-1}) is used to deduce changes in the strength of hydrogen bonding of water in the hydration shell. As expected, we observe cosolvent-induced structural variation in the hydration shell, which has also been reported in the literature[2]. **C)** The area of the peak corresponding to dangling OH (~3670 cm^{-1}) obtained from the three water/cosolvent mixtures indicates that acetone induces the highest number of dangling -OH even though all three have the same amount of total water.

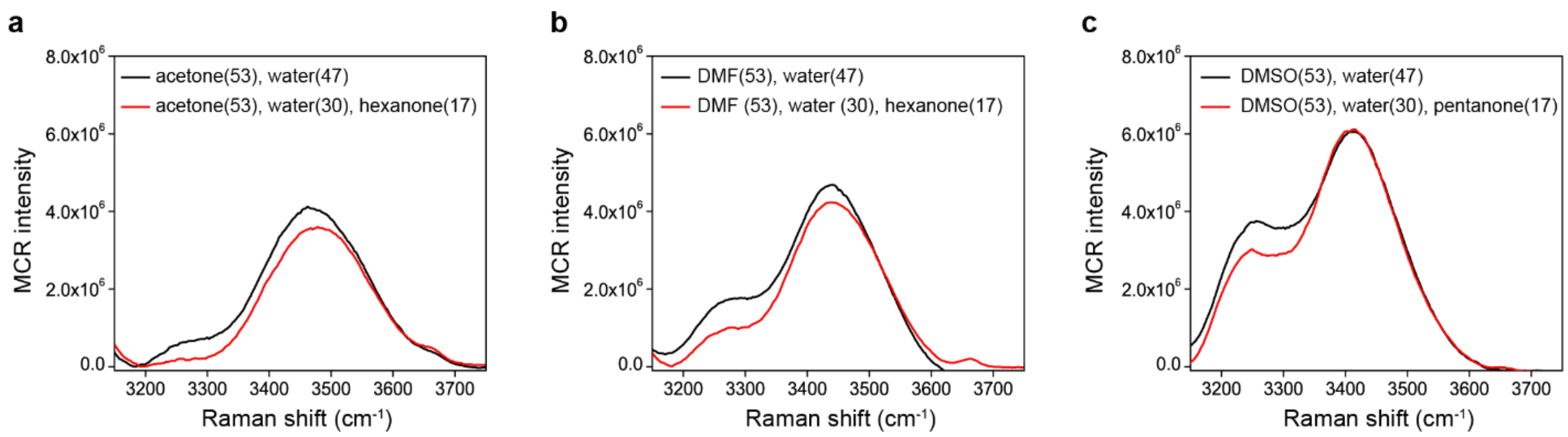

Figure S3. Comparison of Raman MCR spectra between binary water/cosolvent mixtures and ternary water/cosolvent/oil mixtures at matched cosolvent concentration. A) acetone/water/2-hexanone, **B)** DMF/water/2-hexanone, and **C)** DMSO/water/2-pentanone systems. This comparison isolates the effect of oil addition from that of cosolvent dilution: because the cosolvent concentration is held constant between the binary and ternary compositions, decrease in MCR area cannot be attributed to cosolvent removal alone. In all three systems, the ternary MCR area is lower than that of the corresponding binary water/cosolvent mixture, though the difference is smaller for the DMSO/water/2-pentanone system.

3. Supporting tables

Table S1. Molarity measurements for selected compositions of ternary mixtures of oils, cosolvents, and water. The weight of ternary mixtures was measured using a high performance precision balance by OHAUS Scout™ . Density measurements of ternary mixtures were performed with an Anton Paar DMA 1001 densitometer at ~22 °C using precision mode with a standard deviation of ± 0.00002 g/mL. All the measurements were carried out after allowing the solutions to equilibrate to room temperature at ~22 °C. Complete details on the measurement procedure can be found in the Experimental Methods section.

Ternary composition (volume fraction)	Weight (g)	Density (g/cm^3)	Molarity of oil	Molarity of cosolvent	Molarity of water
acetone (70), water (30)	2.559	0.88340	0	9.9	17.2
2-hexanone (5), acetone (65), water (30)	2.562	0.88531	0.4	9.2	17.2
2-hexanone (10), acetone (60), water (30)	2.565	0.88536	0.8	8.4	17.2
2-hexanone (15), acetone (55), water (30)	2.568	0.88399	1.3	7.7	17.2
2-hexanone (17), acetone (53), water (30)	2.569	0.88427	1.4	7.4	17.2
DMF (70), water (30)	2.895	0.99004	0	9.3	17.1
2-hexanone (5), DMF (65), water (30)	2.874	0.98216	0.4	8.7	17.1
2-hexanone (10), DMF (60), water (30)	2.853	0.97555	0.8	8.0	17.1
2-hexanone (15), DMF (55), water (30)	2.832	0.96780	1.3	7.3	17.1
2-hexanone (17), DMF (53), water (30)	2.824	0.96897	1.4	7.0	17.1
DMSO (70), water (30)	3.210	1.09608	0	10.0	16.9
2-pentanone (5), DMSO (65), water (30)	3.165	1.08204	0.5	9.4	17.1
2-pentanone (10), DMSO (60), water (30)	3.122	1.06829	1.0	8.7	17.1

Table S2. Area under the curve of Raman MCR spectra in the O-H stretch region after Gaussian fitting. The total area under the MCR spectrum for the selected compositions was obtained by applying Gaussian fitting to the MCR data in the O-H stretching region using Igor Pro software. The average of three different samples, along with the standard deviation, is reported. Selected compositions were from the single phase region starting from a binary mixture and gradually adding oil at the expense of cosolvent, moving towards the miscibility gap; these compositions are plotted in the phase diagrams of **Figure 2**. In the case of 2-pentanone/DMSO/water, the area of the single-phase region is small as compared to the other two systems, so the compositions with a high volume fraction of oil (15, 17) lie in multiple phase region (empty space in the table below) and have not been analyzed in this work.

	**Acetone/water/2-hexanone**		**DMF/water/2-hexanone**		**DMSO/water/2-pentanone**	
Ternary compositions (volume fraction)	**MCR area (counts cm^{-1})**	Standard deviation	**MCR area (counts cm^{-1})**	Standard deviation	**MCR area (counts cm^{-1})**	Standard deviation
cosolvent (70) water (30)	9.37×10^{8}	1.18×10^{7}	1.05×10^{9}	6.02×10^{6}	1.63×10^{9}	7.41×10^{6}
oil (5) cosolvent (65) water (30)	8.98×10^{8}	1.12×10^{7}	1.01×10^{9}	5.63×10^{6}	1.52×10^{9}	7.65×10^{6}
oil (10) cosolvent (60) water (30)	8.47×10^{8}	1.06×10^{7}	9.75×10^{9}	4.92×10^{6}	1.43×10^{9}	7.60×10^{6}
oil (15) cosolvent (55) water (30)	7.96×10^{8}	1.03×10^{7}	8.87×10^{9}	5.03×10^{6}	-	-
oil (17) cosolvent (53) water (30)	7.67×10^{8}	1.01×10^{7}	8.88×10^{9}	5.12×10^{6}	-	-

Table S3. The area ratio of the 3250 cm^{-1} peak to the 3400 cm^{-1} peak, $A_{3250/3400}$, for three different ternary solvent systems. The area ratio of the 3250 cm^{-1} peak to the 3400 cm^{-1} peak was calculated after fitting the total MCR spectrum into three different peaks (~3250 cm^{-1}, ~3400 cm$^{-1,}$ and ~3600 cm^{-1}). This ratio can be used as a metric to indicate changes in the structure of water in the hydration shell. (Empty spaces correspond to compositions from multiple phase regions of the ternary phase diagram, not covered in this piece of work, as we only target the single-phase region.

	**Acetone/water/2-hexanone**		**DMF/water/2-hexanone**		**DMSO/water/2-pentanone**	
Ternary compositions (volume fraction)	$A_{3250/3400}$	Standard deviation	$A_{3250/3400}$	Standard deviation	$A_{3250/3400}$	Standard deviation
cosolvent (70) water (30)	0.01	0.44 ×10^{-3}	0.12	0.01 ×10^{-2}	0.29	0.65 ×10^{-3}
oil (5) cosolvent (65) water (30)	0.01	0.56 ×10^{-3}	0.11	0.12 ×10^{-2}	0.27	1.08 ×10^{-3}
oil (10) cosolvent (60) water (30)	0.01	0.62 ×10^{-3}	0.10	0.24 ×10^{-2}	0.25	0.56 ×10^{-3}
oil (15) cosolvent (55) water (30)	0.01	0.19 ×10^{-3}	0.09	0.24 ×10^{-2}	-	-
oil (17) cosolvent (53) water (30)	0.01	0.51 ×10^{-3}	0.09	0.28 ×10^{-2}	-	-

Table S4. Viscosity measurements for selected compositions of ternary mixtures of 2-hexanone, acetone, and water. Viscosity measurements were carried out using a Canon-Fenske glass viscometer, which has a cell constant of 0.003570 mm^2/s^2, cSt/s. Prior to measurement, all samples were allowed to equilibrate to room temperature (~22 °C). A detailed description of the viscosity measurement procedure is provided in the Experimental Methods section.

Samples (volume fraction)	Viscosity (cSt)	Density (g/cm^3)	Viscosity (cP)
2-hexanone (15), acetone (55), water (30)	1.37±0.004	0.88399	1.21
2-hexanone (17), acetone (53), water (30)	1.44±0.009	0.88427	1.28

References

(1) Fega, K. R.; Wilcox, A. S.; Ben-Amotz, D. Application of Raman Multivariate Curve Resolution to Solvation-Shell Spectroscopy. *Appl. Spectrosc.* **2012**, *66* (3), 282–288. https://doi.org/10.1366/11-06442.

(2) Ghosh, N.; Roy, S.; Ahmed, M.; Mondal, J. A. Water in the Hydration Shell of Cryoprotectants and Their Non-Cryoprotecting Structural Analogues as Observed by Raman-MCR Spectroscopy. *J. Mol. Liq.* **2018**, *266*, 118–121. https://doi.org/10.1016/j.molliq.2018.06.073.
